# Supplementary material for: Rewiring of the phosphoproteome executes two meiotic divisions in budding yeast
Source: EMBO J. 2024 Feb 27;43(7):11. doi: 10.1038/s44318-024-00059-8 (PMC10987667; doi:10.1038/s44318-024-00059-8)
Supplement: Supplementary file 2 — Appendix [file 44318_2024_59_MOESM2_ESM.pdf]

Appendix for:

Lori B. Koch[1], Christos Spanos[1], Van Kelly[1], Tony Ly[1, 2] and Adele L. Marston[1]*

Rewiring of the phosphoproteome executes two meiotic divisions

Contents

Appendix Figure S1………..Page 2
Appendix Figure S2………..Page 4
Appendix Figure S3………..Page 5
Appendix Figure S4………..Page 7
Appendix Figure S5………..Page 8
Appendix Figure S6………..Page 9
Appendix Figure S7………..Page 10
Appendix Figure S8………..Page 11
Appendix Figure S9………..Page 12
Appendix Figure S10………Page 14
Appendix Figure S11………Page 16
Appendix Figure S12………Page 18

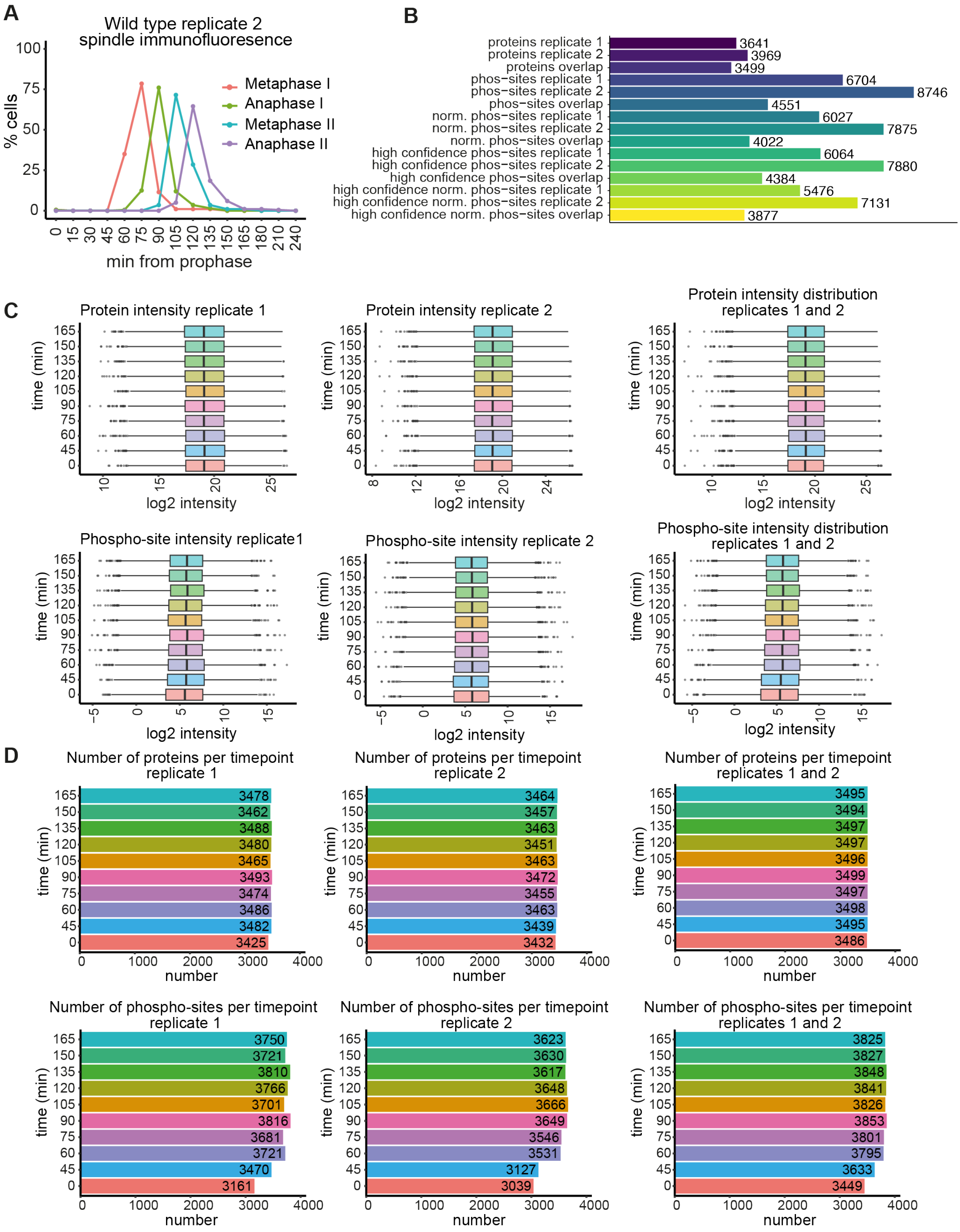

Appendix Figure S1 A high quality wild type meiotic proteomic and phospho-proteomic timecourse dataset.

A. Morphology of the spindle in the wild type replicate 2 meiotic timecourse. Time indicates minutes after prophase release.

B. Total numbers of unique proteins and phospho-sites detected for the replicate wild type timecourses. “Norm.” indicates normalized, meaning phospho-site abundance is normalized to corresponding protein level. High confidence indicates localization probability > 0.75.
C. Protein and phospho-site intensity distributions for individual replicates (left two columns) and the combined two-experiment dataset (right column).
D. Protein and phospho-site numbers detected at each time-point for individual replicates (left two columns) and the combined two-experiment dataset (right column).

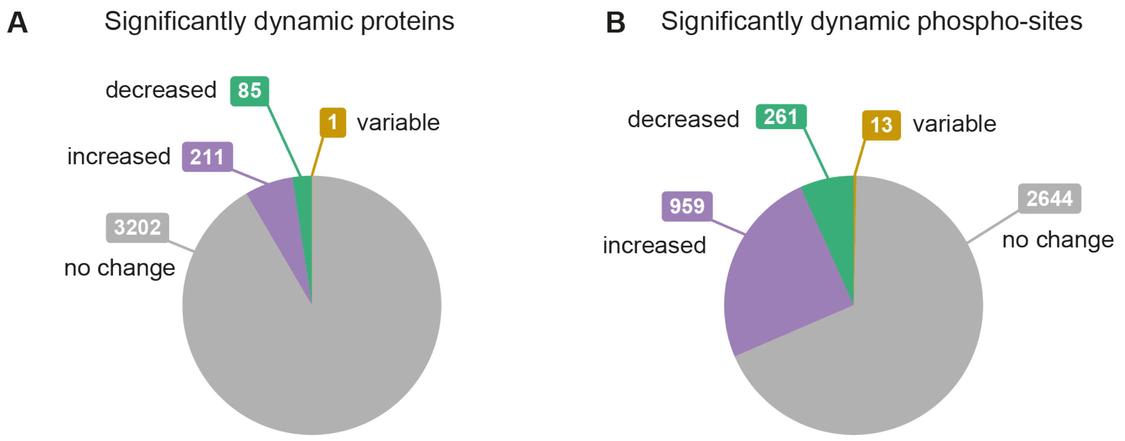

Appendix Figure S2 A greater fraction of phospho-sites than proteins are dynamic.

A. Proportion of proteins that significantly increased, decreased or had a variable trend from time zero. See Appendix Table S1 for protein identities.

B. Proportion of phospho-sites that significantly increased, decreased or had a variable trend from time zero. See Appendix Table S3 for list of phospho-sites in each category.

A

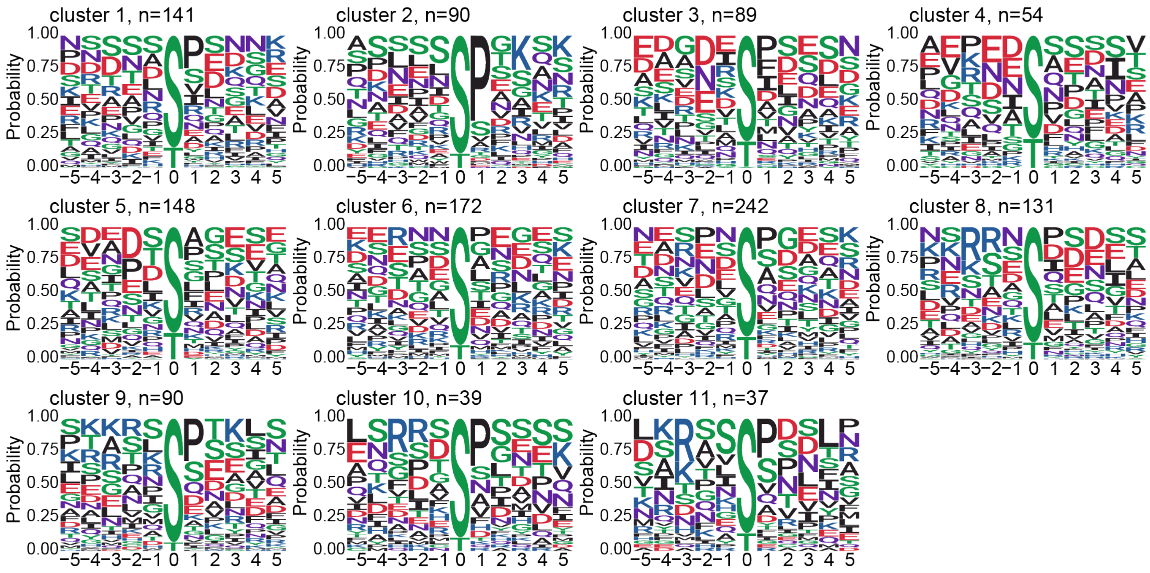

B

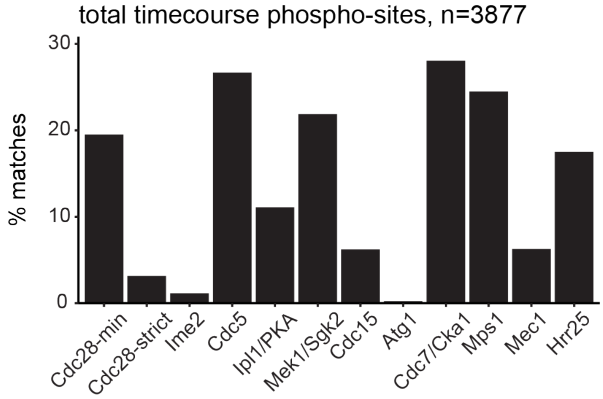

C

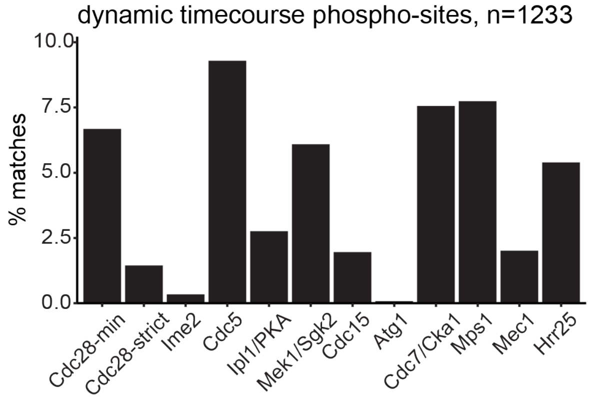

Appendix Figure S3 Motif enrichment analysis of phospho-sites across the meiotic divisions timecourse.

A. Motif logos of the 11 phospho-site clusters presented in Fig 3 and EV2.

B. Bar graph of the percent of motif matching sites for each of the 12 selected kinase motifs in the total timecourse dataset n=3877.

C. Bar graph of the percent of motif matching sites for each of the 12 selected kinase motifs in the sites that were dynamic from time zero (including all 11 clusters from Fig 3 and EV2) n=1233.

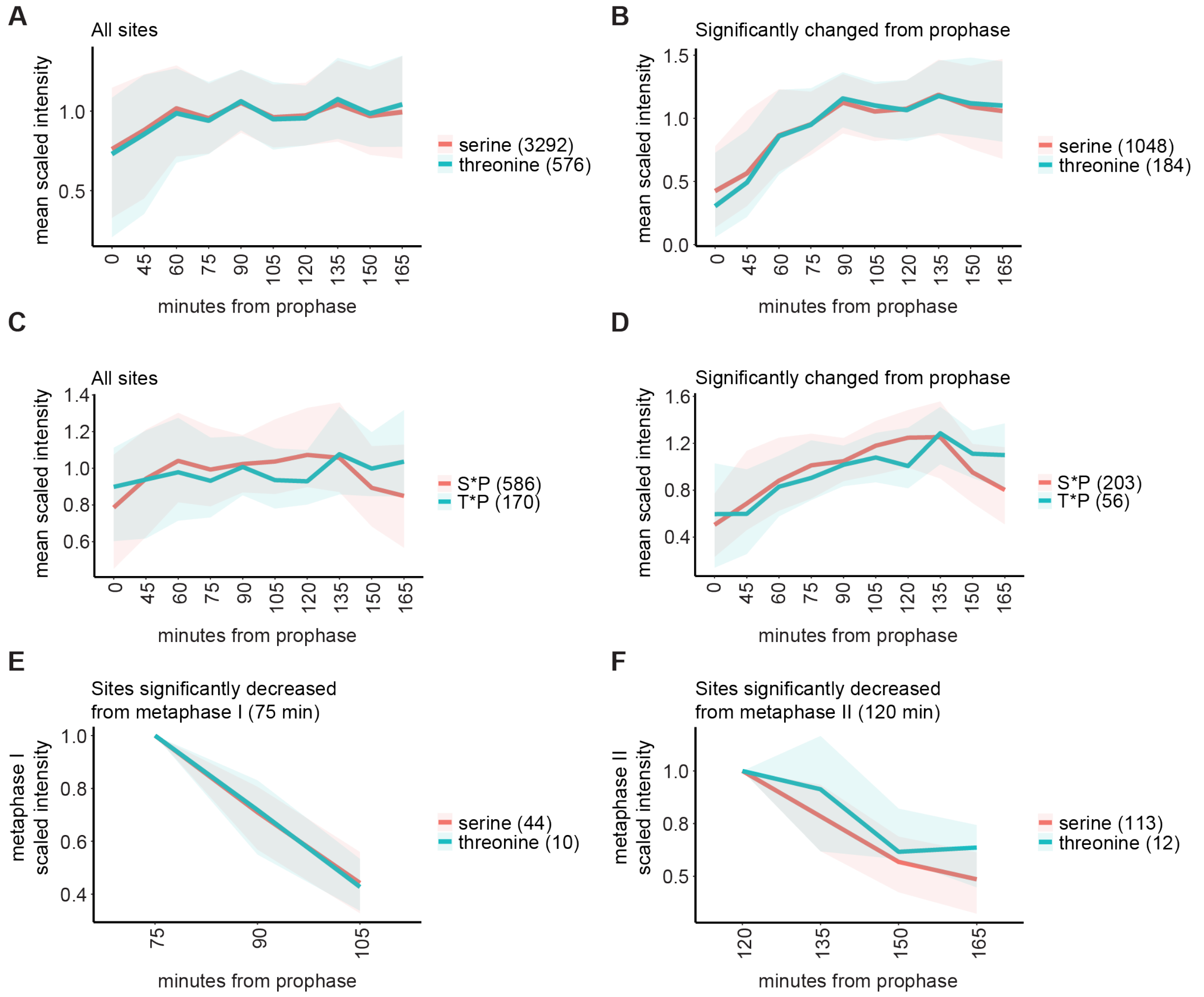

Appendix Figure S4 Kinetics of the changes in serine and threonine phosphorylation during the meiotic divisions.

A. Scaled median abundance of serine or threonine-directed phosphorylation for all sites in the wild type timecourse.

B. Scaled median abundance of serine or threonine-directed phosphorylation sites that significantly change in abundance from time zero in the wild type timecourse.

C. As for A but including only those phospho-sites that the $Cdc28^{Cdk1}$ minimal consensus [ST]*P.

D. As for B but including only those phospho-sites that match the $Cdc28^{Cdk1}$ minimal consensus [ST]*P.

E. Scaled median abundance for serine or threonine phospho-sites that significantly decrease in abundance after metaphase I (75 min). Scaled to metaphase I abundance.

F. Scaled median abundance for serine or threonine phospho-sites that significantly decrease in abundance after metaphase II (120 min). Scaled to metaphase II abundance.

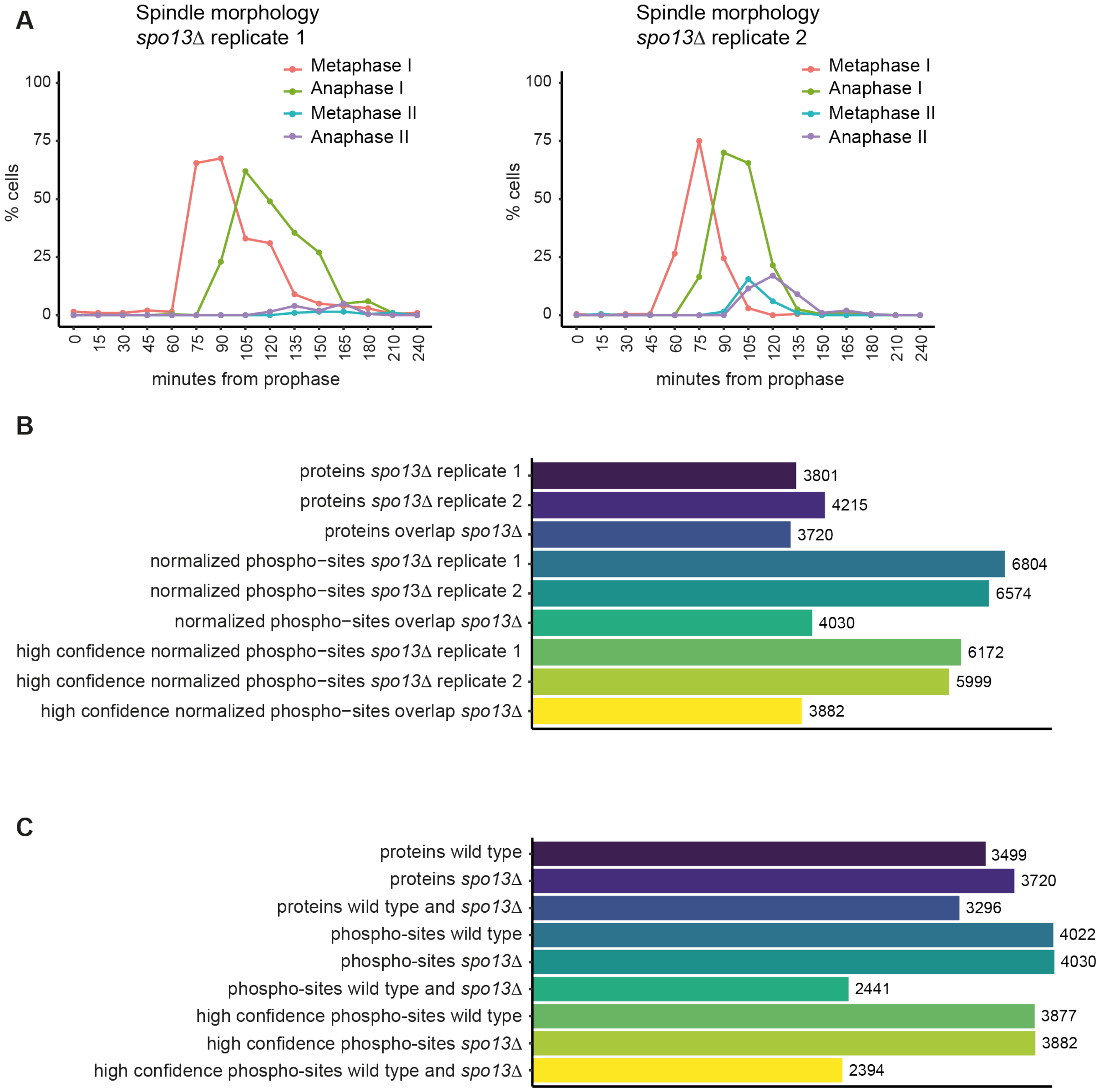

Appendix Figure S5 The time-resolved phosphoproteome of the *spo13Δ* mutant.

A. Morphology of the spindle in each biological replicate of *spo13Δ* timecourses illustrates the level of synchrony and the single meiotic division of these cells.

B. Total numbers of unique proteins and phospho-sites detected in *spo13Δ* timecourses. Normalized indicates phospho-site abundance is normalized to corresponding protein level. High confidence indicates localization probability > 0.75.

C. Total numbers of unique proteins and phospho-sites detected in the combined wild type and *spo13Δ* dataset. All phospho-site numbers in this plot indicate phospho-sites normalized to corresponding protein level. High confidence indicates localization probability > 0.75.

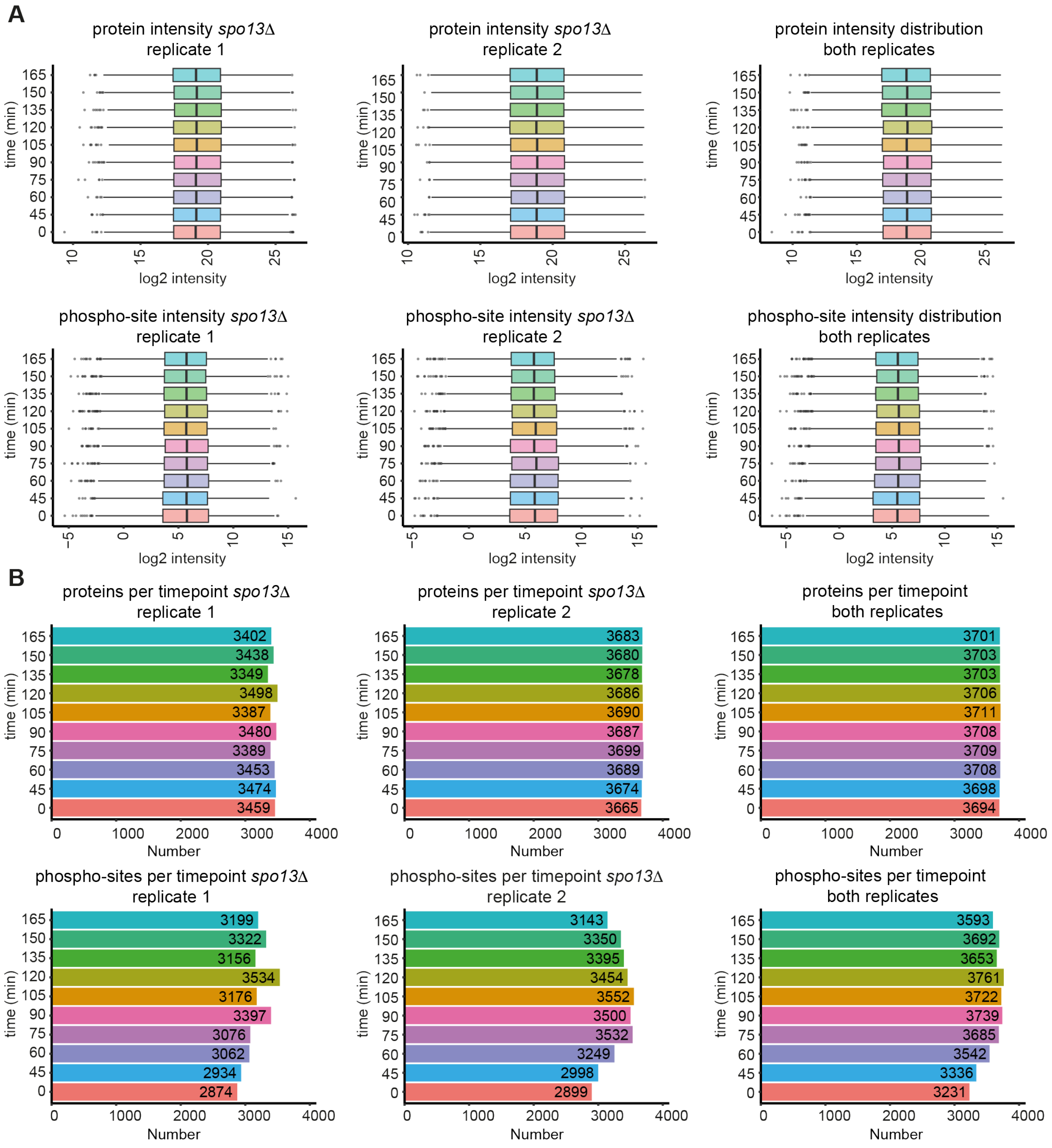

Appendix Figure S6 Protein and phospho-site intensity distributions and number of proteins detected per timepoint for the *spo13Δ* dataset.

A. Protein and phospho-site intensity distributions for individual replicates (left two columns) and the combined two-experiment dataset (right column).

B. Protein and phospho-site number detected at each timepoint in individual replicates (left two columns) and the combined two-experiment dataset (right column).

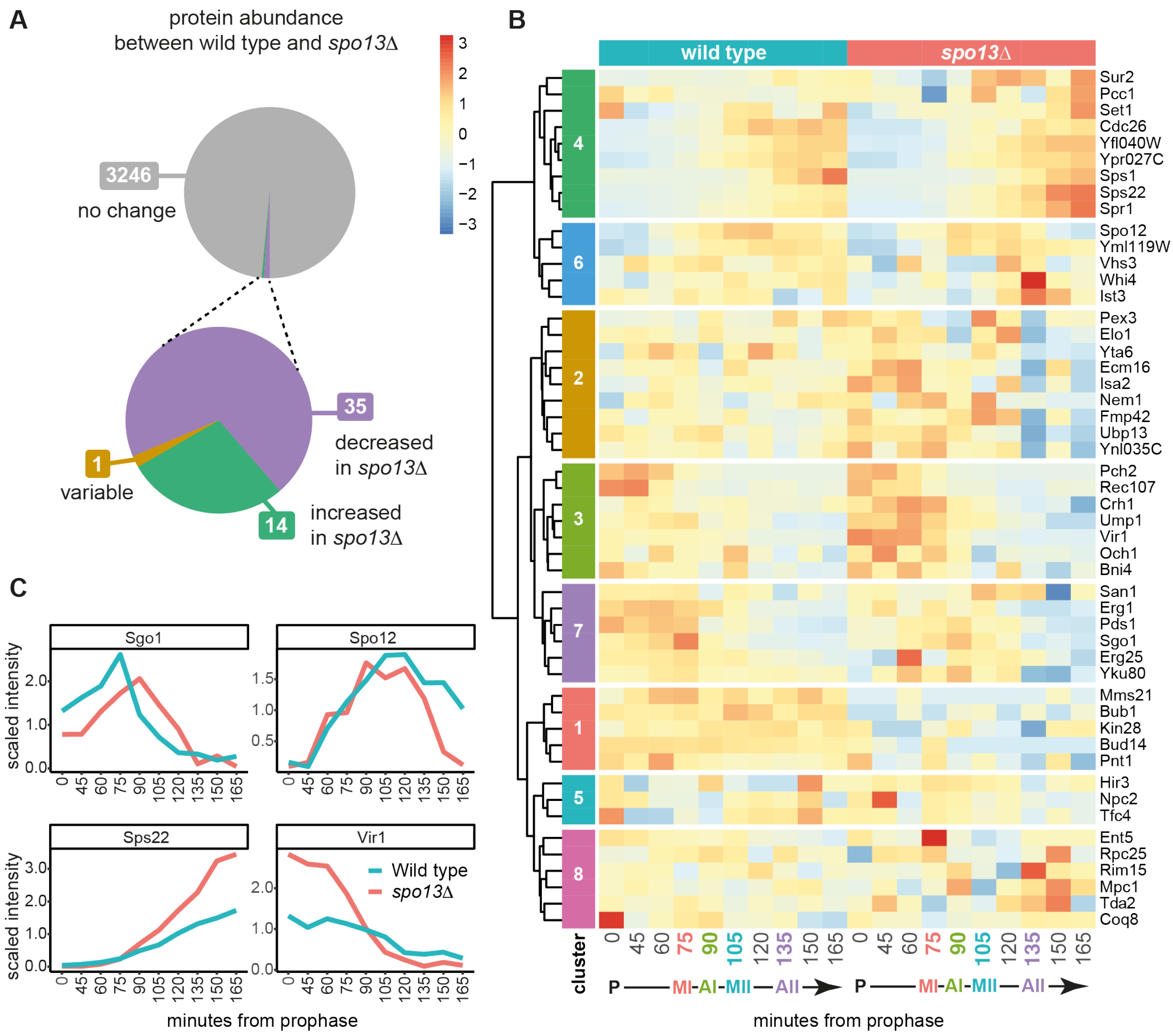

Appendix Figure S7 Proteins with significantly different abundance in wild type versus *spo13Δ* cells*.*

A. Proportion of proteins which significantly vary between wild type and *spo13Δ* at matched time points. See also Appendix Table S5.

B. Hierarchical clustering of significantly different proteins, n=50, between wild type and *spo13Δ* across the meiotic divisions. See also Appendix Table S6.

C. Median abundances of selected proteins that significantly differ between wild type and *spo13Δ* from B.

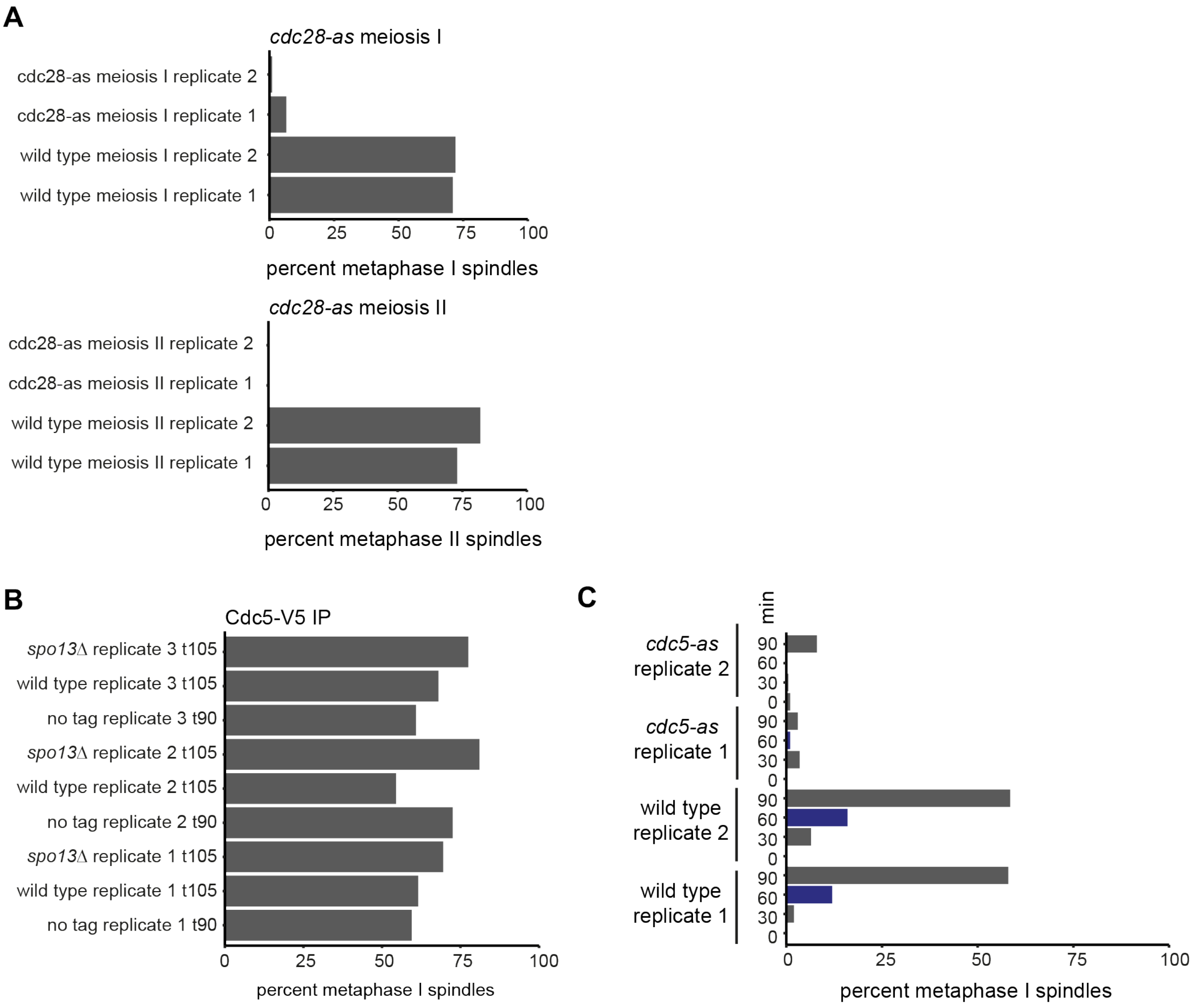

Appendix Figure S8 Spindle morphology in the *cdc28-as*, Cdc5 IP and *cdc5-as* experiments

A. For the *cdc28-as* experiment, the percent of cells with metaphase I spindles in the ‘meiosis I’ samples (top) or metaphase II spindles in the ‘meiosis II’ samples (bottom).
B. For the Cdc5-V5 IP experiment, the percent of cells with metaphase I spindles in each sample. The time in minutes after prophase release is indicated for example as t90.
C. For the *cdc5-as* experiment, the percent of cells with metaphase I spindles by spindle immunofluorescence. Blue bars indicate the timepoint at which samples for mass spectrometry were collected (60 min). Minutes are after prophase arrest.

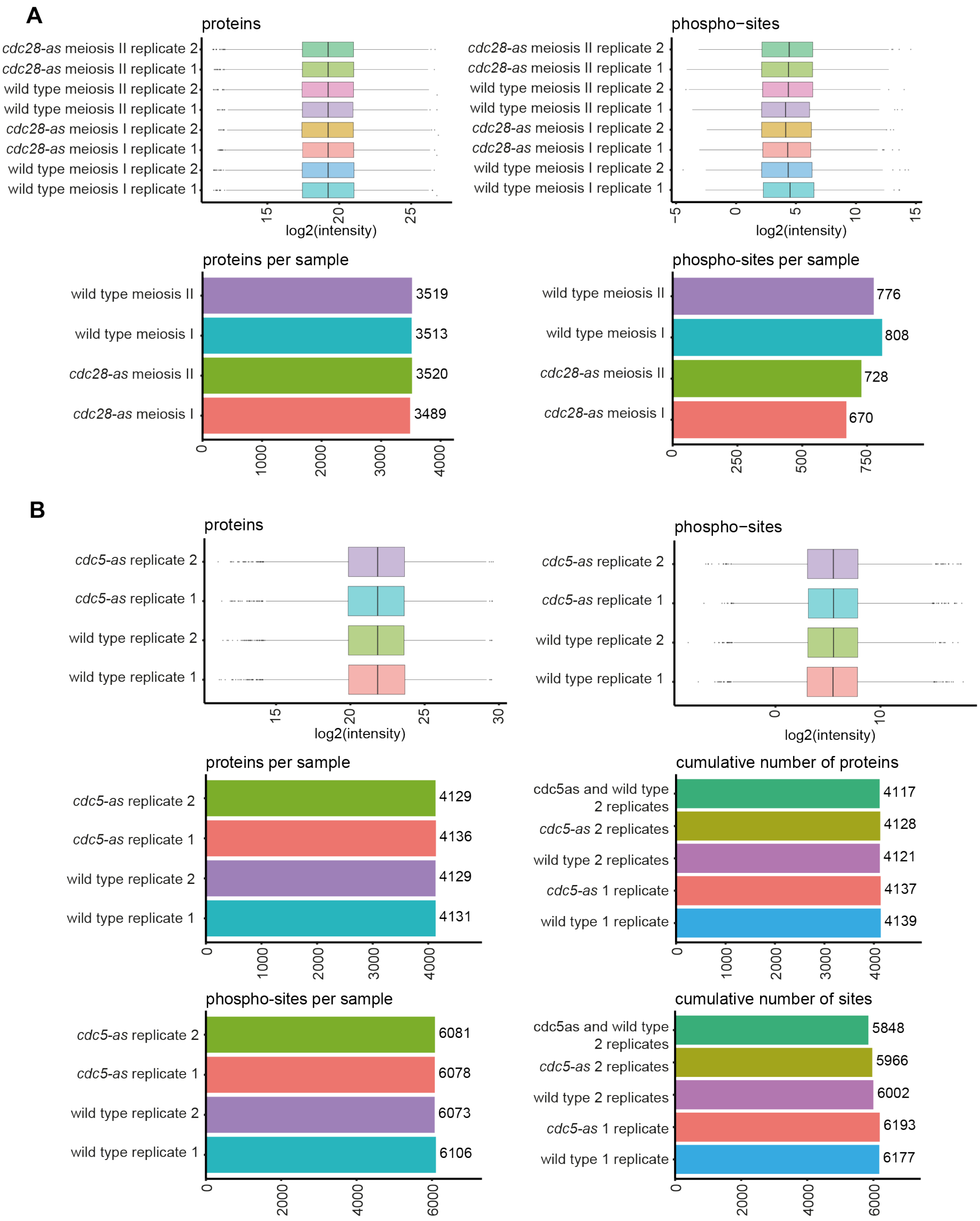

Appendix Figure S9 Distribution and number of proteins and phospho-sites detected in the *cdc28-as* and *cdc5-as* experiments

A. (top, left) Distribution of protein intensities in each sample in the wild type vs *cdc28-as* experiment.

   (top, right) Distribution of phospho-site intensities in each sample in the wild type vs *cdc28-as* experiment.

(bottom, left) Number of proteins found in at least 1 out of 2 replicates of each sample.

(bottom, right) Number of phospho-sites found in at least 1 out of 2 replicates of each sample.

B. (top, left) Distribution of protein intensities in each sample in the wild-type vs *cdc5-as* in early metaphase I experiment.

   (top, right) Distribution of phospho-site intensities in each sample in the wild-type vs *cdc5-as* in early metaphase I experiment.

   (middle, left) Number of proteins found in each sample in the wild-type vs *cdc5-as* in early metaphase I experiment.

   (middle, right) Cumulative number of proteins detected in the indicated groups.

   (bottom, left) Number of phospho-sites found in each sample in the wild-type vs *cdc5-as* in early metaphase I experiment.

   (bottom, right) Cumulative number of phospho-sites detected in the indicated groups.

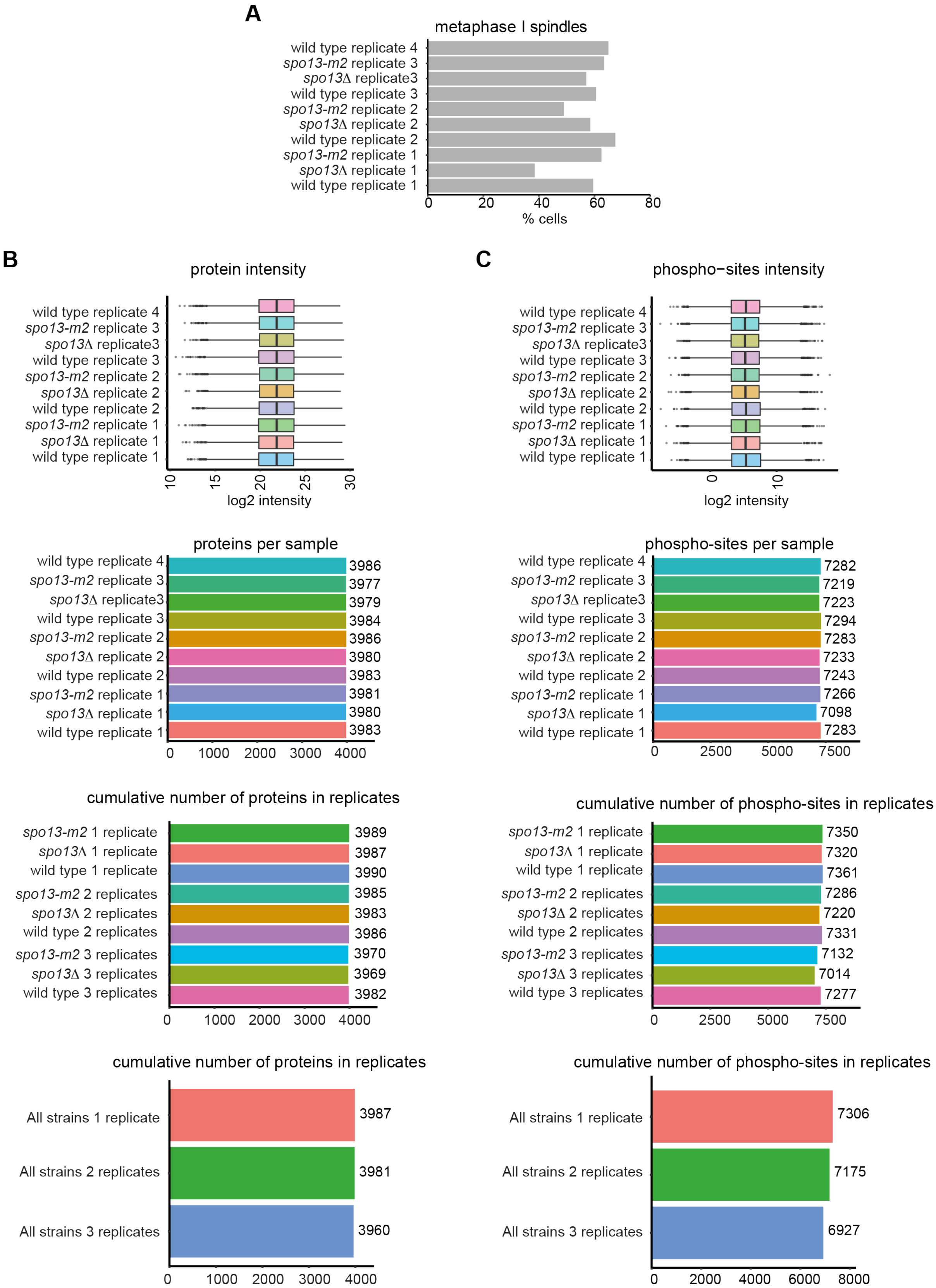

Appendix Figure S10 Quality control of metaphase I arrest proteomics and phospho-proteomics.

A. Percentage of cells with metaphase I spindle morphology.

B. Protein intensity distribution and number of detections per sample.

C. Phospho-site intensity distribution and number of detections per sample.

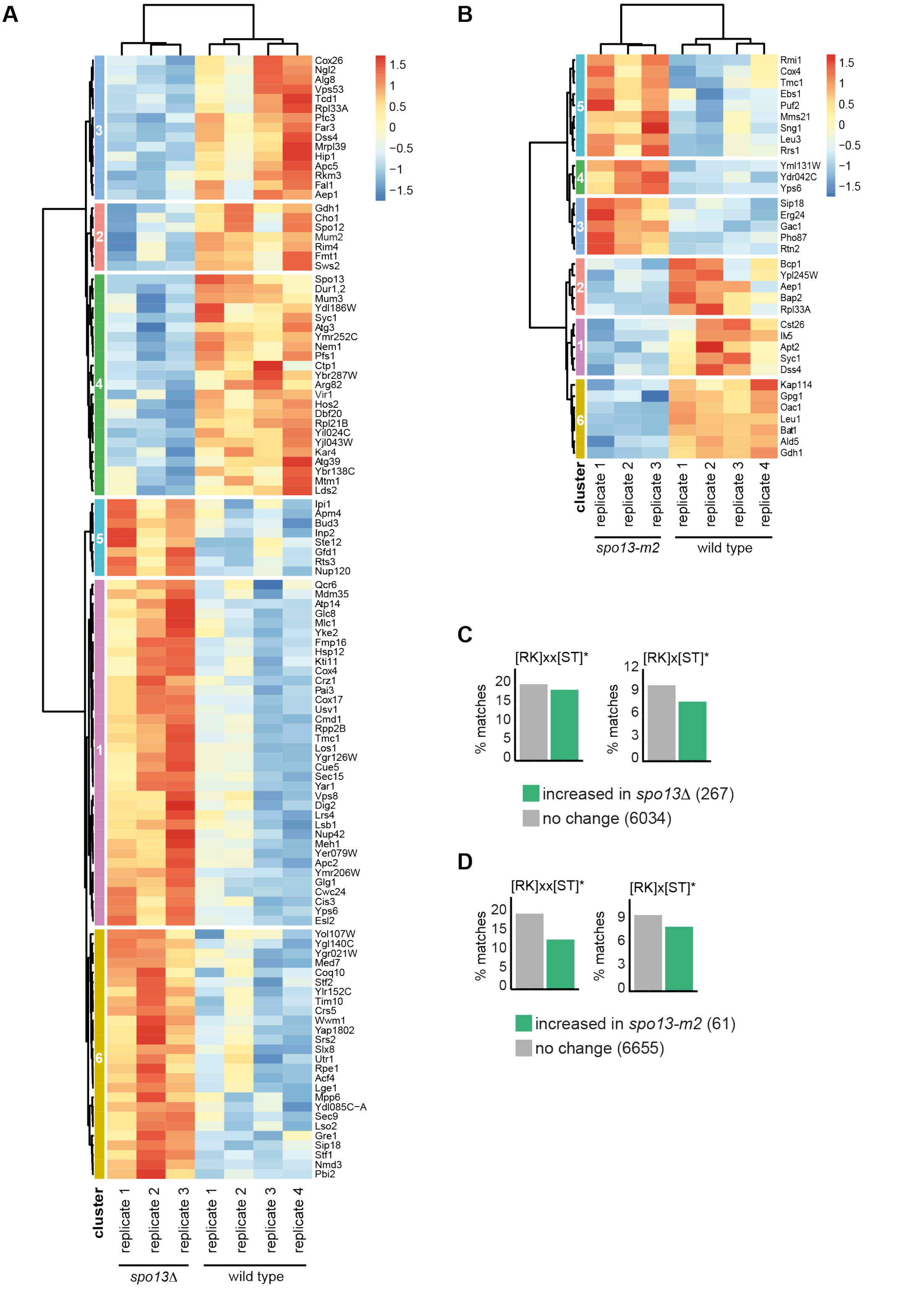

Appendix Figure S11 Metaphase I arrest protein clustering analysis and phospho-site motif analysis

A. Clustering of proteins with significantly different abundance between wild type and *spo13*Δ in metaphase I arrested cells.
B. Clustering of proteins with significantly different abundance between wild type and *spo13-m2* in metaphase I arrested cells.
C. Percent motif matches in the indicated groups of sites, as shown in the pie charts in Figure 7. Fisher's exact tests were carried out for each comparison but none were significant (p-value < 0.05).

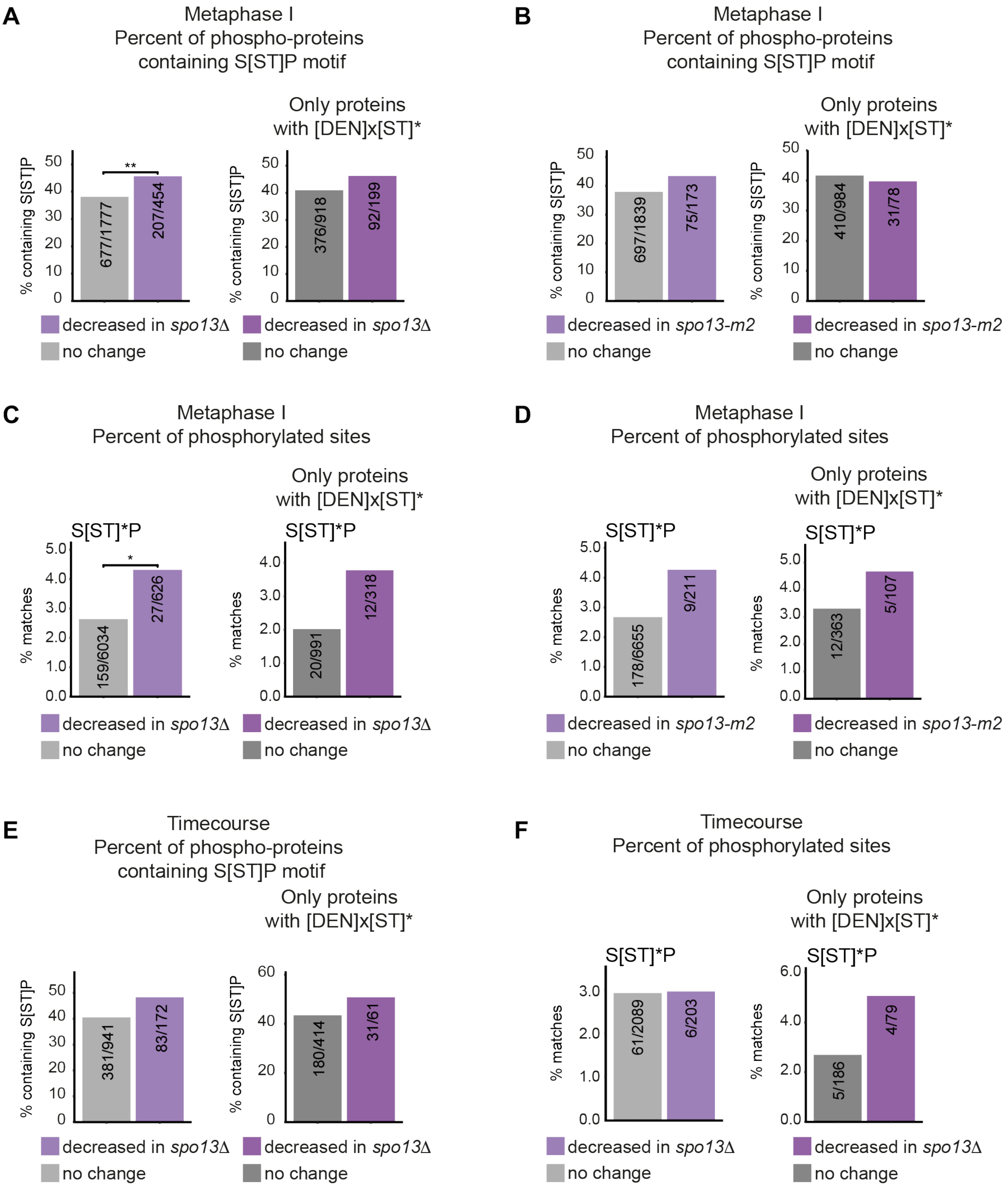

Appendix Figure S12 Analysis of the polo-box binding S[ST]P motif in *spo13* mutants.

A. (Left) Fisher test comparing the fraction of proteins containing the S[ST]P motif at any position among proteins with decreased phosphorylation in metaphase I *spo13*Δ versus no significant difference in phosphorylation in *spo13*Δ. (Right) The same but only between the subset of each group from the left where there was at least one phosphorylation on a [DEN]x[ST]* motif site. Asterisks represent p-value ($p<0.001$ = ***, $p<0.01$ = **, $p<0.05$ = *).

B. The same as A but comparing *spo13-m2* and wild type metaphase I samples. No comparisons were significant with p-value < 0.05.
C. (Left) Fisher test comparing the number of sites with phosphorylation on the S[ST]*P motif between sites with decreased phosphorylation in metaphase I *spo13*Δ versus no change. (Right) The same but only between the subset of proteins included in the groups from the left where there was at least one phosphorylation on a [DEN]x[ST]* motif site. Asterisks represent p-value (p<0.001 = ***, p<0.01 = **, p<0.05 = *).
D. The same as C but comparing *spo13-m2* and wild type metaphase I samples. No comparisons were significant with p-value < 0.05.
E. The same as A but analysing phospho-proteins with decreased phosphorylation in *spo13*Δ versus wild type at any time during the full meiotic timecourse. No comparisons were significant with p-value < 0.05.
F. The same as C but analysing phosphorylated sites decreased in *spo13*Δ versus wild type at any time during the full meiotic timecourse. No comparisons were significant with p-value < 0.05.
