# Supplementary figures and images for: Rewiring of the phosphoproteome executes two meiotic divisions in budding yeast
Source: EMBO J. 2024 Feb 27;43(7):11. doi: 10.1038/s44318-024-00059-8 (PMC10987667; doi:10.1038/s44318-024-00059-8)

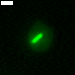

Supplement: Supplementary file 18 — Source Data Fig. 1 [file 44318_2024_59_MOESM18_ESM.zip › Source Data Figure 1/1A/metaphaseI_GFP-1_green_scalebar.png]

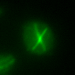

Supplement: Supplementary file 18 — Source Data Fig. 1 [file 44318_2024_59_MOESM18_ESM.zip › Source Data Figure 1/1A/anaphaseII_GFP-1_green.png]

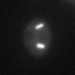

Supplement: Supplementary file 18 — Source Data Fig. 1 [file 44318_2024_59_MOESM18_ESM.zip › Source Data Figure 1/1A/metaphaseII_GFP-1.png]

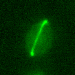

Supplement: Supplementary file 18 — Source Data Fig. 1 [file 44318_2024_59_MOESM18_ESM.zip › Source Data Figure 1/1A/anaphaseI_GFP-1_green.png]

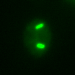

Supplement: Supplementary file 18 — Source Data Fig. 1 [file 44318_2024_59_MOESM18_ESM.zip › Source Data Figure 1/1A/metaphaseII_GFP-1_green.png]

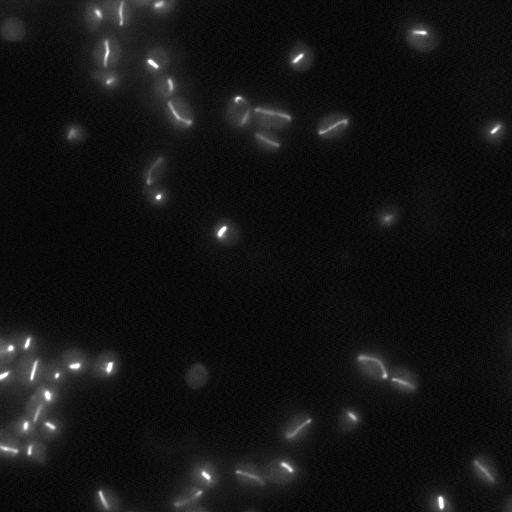

Supplement: Supplementary file 18 — Source Data Fig. 1 [file 44318_2024_59_MOESM18_ESM.zip › Source Data Figure 1/1A/metaphaseI_GFP.tif]

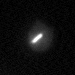

Supplement: Supplementary file 18 — Source Data Fig. 1 [file 44318_2024_59_MOESM18_ESM.zip › Source Data Figure 1/1A/metaphaseI_GFP-1.png]

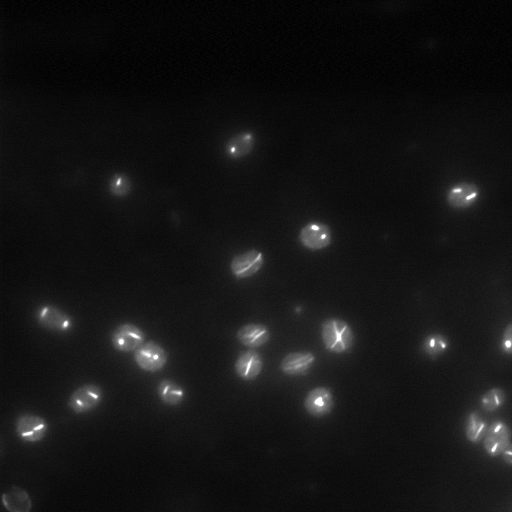

Supplement: Supplementary file 18 — Source Data Fig. 1 [file 44318_2024_59_MOESM18_ESM.zip › Source Data Figure 1/1A/anaphaseII_GFP_MMStack_Pos0.ome.tif]

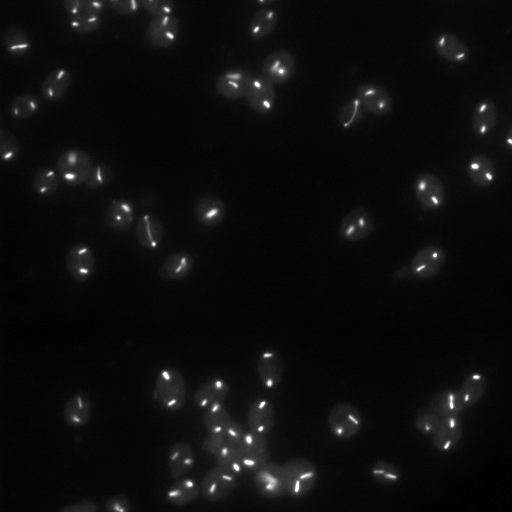

Supplement: Supplementary file 18 — Source Data Fig. 1 [file 44318_2024_59_MOESM18_ESM.zip › Source Data Figure 1/1A/metaphaseII_GFP_MMStack_Pos0.ome.tif]

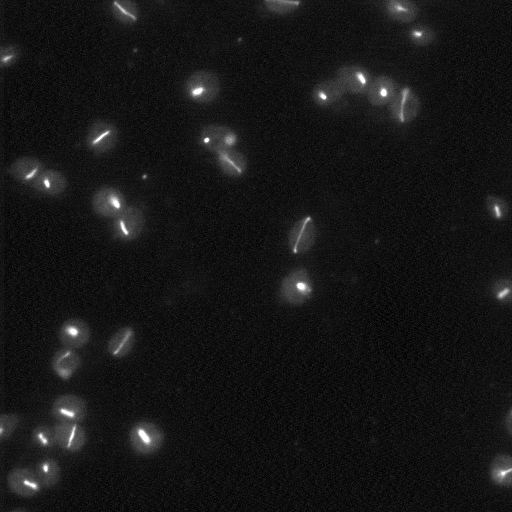

Supplement: Supplementary file 18 — Source Data Fig. 1 [file 44318_2024_59_MOESM18_ESM.zip › Source Data Figure 1/1A/anaphaseI_GFP_MMStack_Pos0.ome.tif]

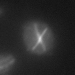

Supplement: Supplementary file 18 — Source Data Fig. 1 [file 44318_2024_59_MOESM18_ESM.zip › Source Data Figure 1/1A/anaphaseII_GFP-1.png]

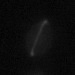

Supplement: Supplementary file 18 — Source Data Fig. 1 [file 44318_2024_59_MOESM18_ESM.zip › Source Data Figure 1/1A/anaphaseI_GFP-1.png]
