# Supplementary material for: Rewiring of the phosphoproteome executes two meiotic divisions in budding yeast
Source: EMBO J. 2024 Feb 27;43(7):11. doi: 10.1038/s44318-024-00059-8 (PMC10987667; doi:10.1038/s44318-024-00059-8)
Supplement: Supplementary file 29 — Expanded View Figures [file 44318_2024_59_MOESM29_ESM.pdf]

Expanded View Figures

Figure EV1. Protein dynamics at the metaphase-to-anaphase transition in meiosis I and II.

(**A**) Fold change in protein abundance of proteins that significantly change from metaphase I to anaphase I. (**B**) Fold change in protein abundance of proteins that significantly change from metaphase II to anaphase II. (**C**) GO term analysis of proteins from (**A**). Data information: Statistics: Cumulative hypergeometric test followed by correction for multiple testing (gprofiler2 R package gost function default settings). (**D**) GO term analysis of proteins from (**B**). Data information: Statistics: Cumulative hypergeometric test followed by correction for multiple testing (gprofiler2 R package gost function default settings). Source data are available online for this figure.

© The Author(s)

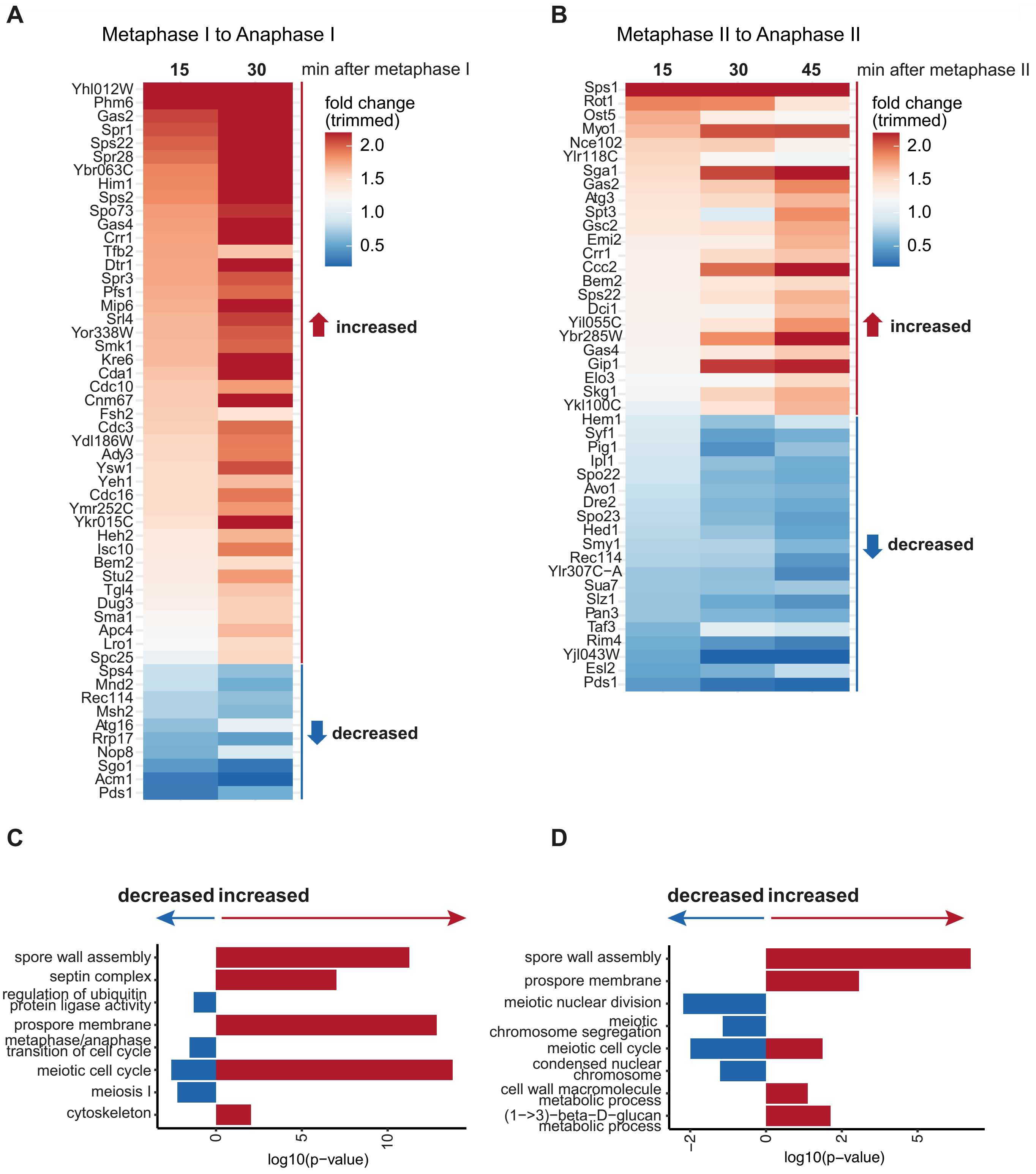
A
Metaphase I to Anaphase I
15
30
min after metaphase I
Yhl012W
Phm6
Gas2
Spr1
Sps22
Spr28
Ybr063C
Him1
Sps2
Spo73
Gas4
Crr1
Tfb2
Dtr1
Spr3
Pfs1
Mip6
Srl4
Yor338W
Smk1
Kre6
Cda1
Cdc10
Cnm67
Fsh2
Cdc3
Ydl186W
Ady3
Ysw1
Yeh1
Cdc16
Ymr252C
Ykr015C
Heh2
Isc10
Bem2
Stu2
Tgl4
Dug3
Sma1
Apc4
Lro1
Spc25
Sps4
Mnd2
Rec114
Msh2
Atg16
Rrp17
Nop8
Sgo1
Acm1
Pds1
fold change
(trimmed)
2.0
1.5
1.0
0.5
increased
decreased
B
Metaphase II to Anaphase II
15
30
45
min after metaphase II
Sps1
Rot1
Ost5
Myo1
Nce102
Ylr118C
Sga1
Gas2
Atg3
Spt3
Gsc2
Emi2
Crr1
Ccc2
Bem2
Sps22
Dci1
Yil055C
Ybr285W
Gas4
Gip1
Elo3
Skg1
Ykl100C
Hem1
Syf1
Pig1
Ipl1
Spo22
Avo1
Dre2
Spo23
Hed1
Smy1
Rec114
Ylr307C−A
Sua7
Slz1
Pan3
Taf3
Rim4
Yjl043W
Esl2
Pds1
fold change
(trimmed)
2.0
1.5
1.0
0.5
increased
decreased
C
decreased increased
spore wall assembly
septin complex
regulation of ubiquitin protein ligase activity
prospore membrane
metaphase/anaphase transition of cell cycle
meiotic cell cycle
meiosis I
cytoskeleton
0
5
10
log10(p−value)
D
decreased increased
spore wall assembly
prospore membrane
meiotic nuclear division
meiotic chromosome segregation
meiotic cell cycle
condensed nuclear chromosome
cell wall macromolecule metabolic process
(1−>3)−beta−D−glucan metabolic process
−2
0
2
5
log10(p−value)

 © The Author(s)

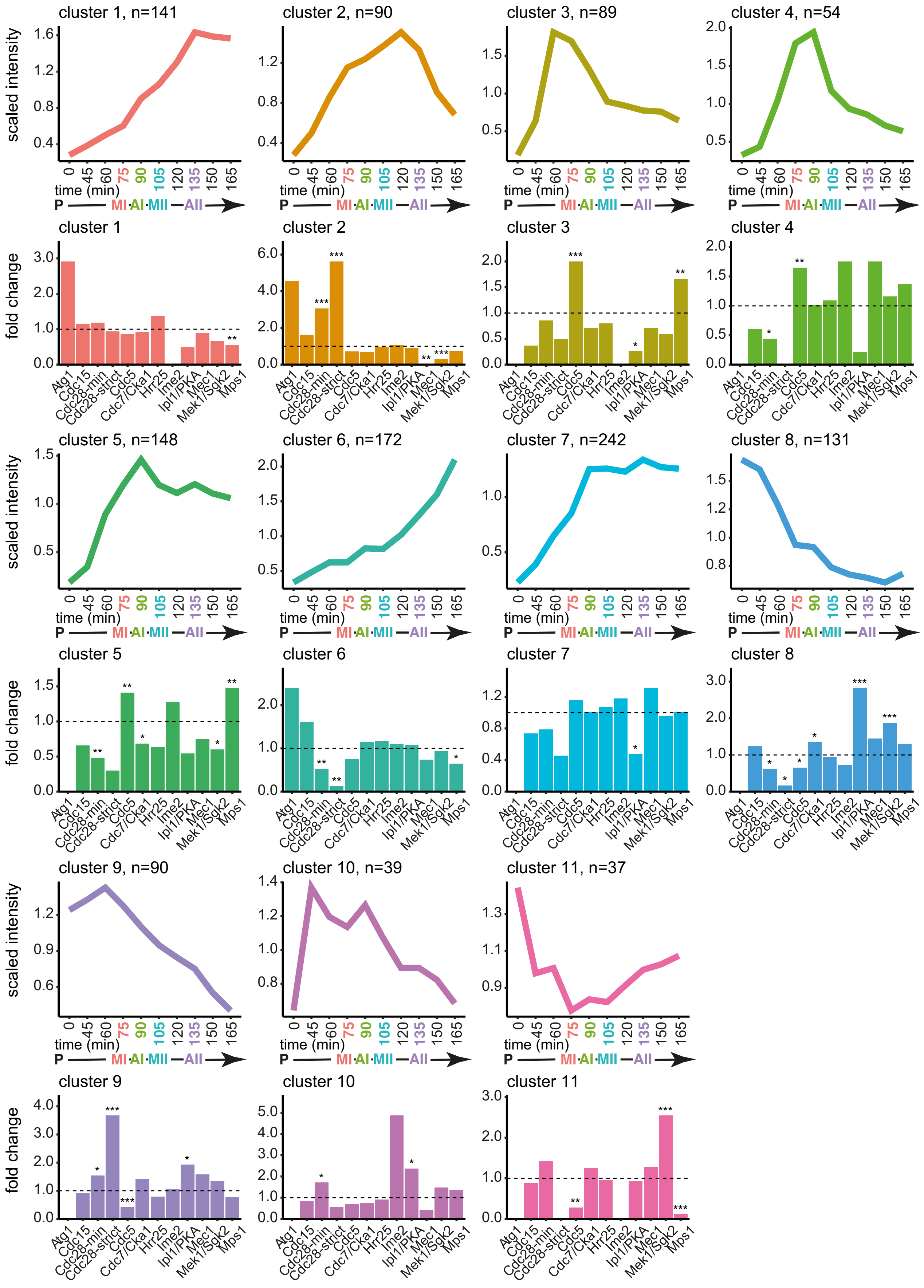
cluster 1, n=141
cluster 2, n=90
cluster 3, n=89
cluster 4, n=54
cluster 5, n=148
cluster 6, n=172
cluster 7, n=242
cluster 8, n=131
cluster 9, n=90
cluster 10, n=39
cluster 11, n=37
scaled intensity
time (min)
0 45 60 75 90 105 120 135 150 165
P MI·AI·MII AII
cluster 1
cluster 2
cluster 3
cluster 4
cluster 5
cluster 6
cluster 7
cluster 8
cluster 9
cluster 10
cluster 11
fold change
Atg1 Cdc15 Cdc28-min Cdc28-strict Cdc5 Cdc7/Cka1 Hrr25 Ime2 Ipl1/PKA Mec1 Mek1/Sgk2 Mps1

Figure EV2. Enrichment of kinase consensus motifs for clusters of dynamic phosphorylation sites.

Median lineplots of all clusters from Fig. 3A and kinase motif enrichment analysis bar graphs. Asterisks represent p value from Fisher's exact test $^{**}P < 0.01$; $^{*}P < 0.05$). Source data are available online for this figure.

 © The Author(s)

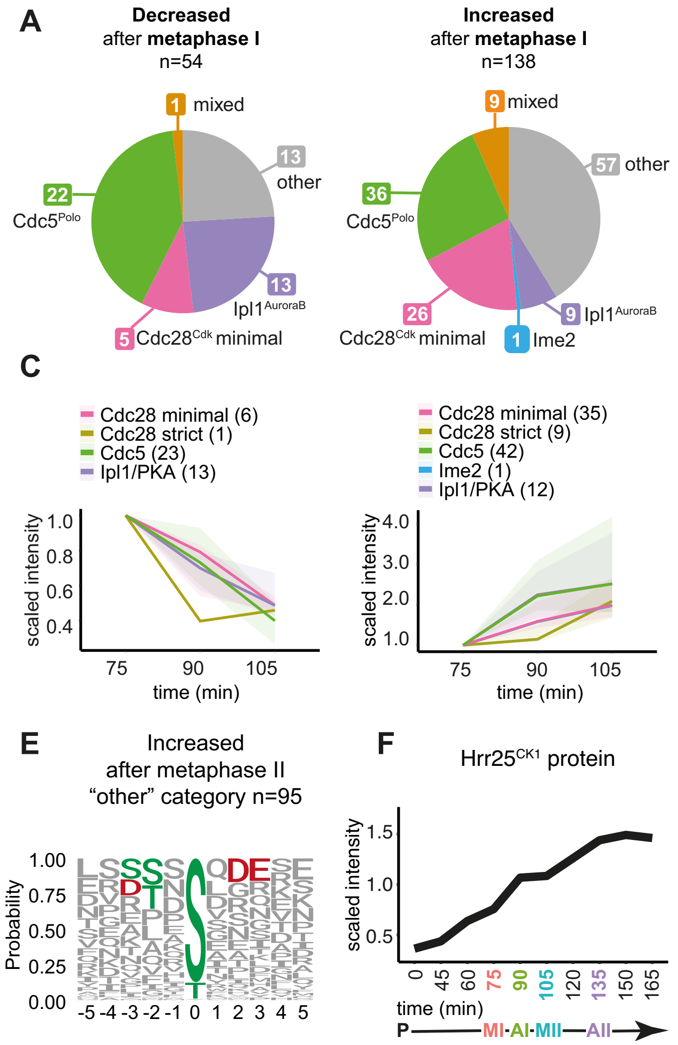

Figure EV3. Phosphorylation dynamics at the metaphase-to-anaphase transitions in meiosis I and II.

(**A**) Motifs matching phospho-sites that significantly decrease (left) or increase (right) at the metaphase I to anaphase I transition. (**B**) Motifs matching phospho-sites that significantly decrease (left) or increase (right) at the metaphase II to anaphase II transition. (**C**) Median change of motif-matching phospho-sites decreasing (left) or increasing (right) from metaphase I to anaphase I. Abundance scaled to 75 min (metaphase I). (**D**) Median change of motif-matching phospho-sites decreasing (left) or increasing (right) from metaphase II to anaphase II. Abundance scaled to 120 min (metaphase II). (**E**) Motif logo of phospho-sites that are increased after metaphase II from (**B**) (right), which do not match any of the selected motifs, from the "other" category $n = 95$. (**F**) Abundance of Hrr25^{CK1} protein rises in meiosis II. Source data are available online for this figure.

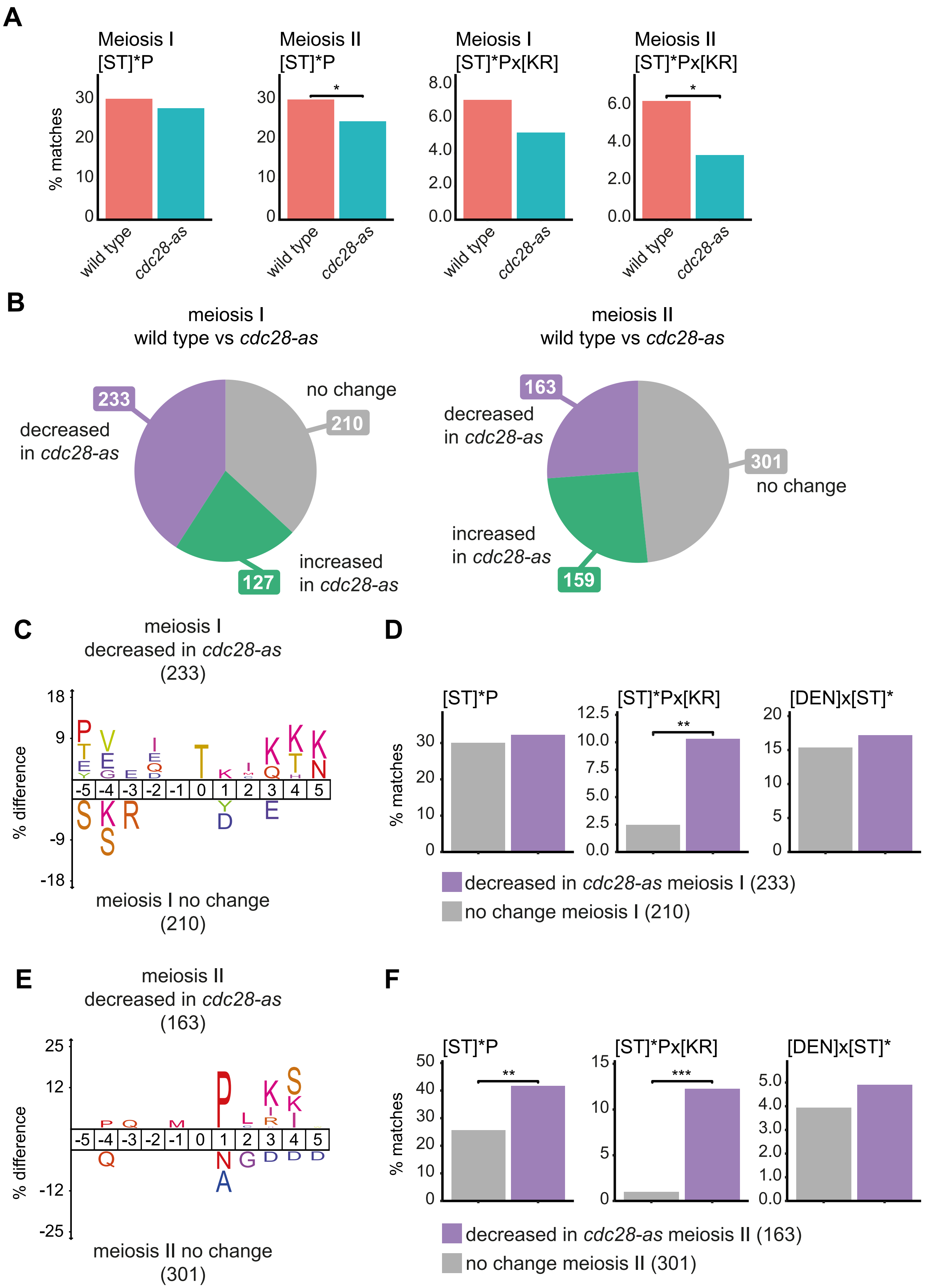
A
Meiosis I [ST]*P
Meiosis II [ST]*P
Meiosis I [ST]*Px[KR]
Meiosis II [ST]*Px[KR]
% matches
wild type
cdc28-as
B
meiosis I wild type vs cdc28-as
meiosis II wild type vs cdc28-as
233
decreased in cdc28-as
no change
210
127
increased in cdc28-as
163
301
159
C
meiosis I decreased in cdc28-as (233)
meiosis I no change (210)
% difference
D
[ST]*P
[ST]*Px[KR]
[DEN]x[ST]*
decreased in cdc28-as meiosis I (233)
no change meiosis I (210)
E
meiosis II decreased in cdc28-as (163)
meiosis II no change (301)
F
decreased in cdc28-as meiosis II (163)
no change meiosis II (301)

 © The Author(s)

Figure EV4. Strict Cdk consensus site phosphorylation is the best predictor of Cdc28^{Cdk1} kinase activity.

(**A**) Fisher tests comparing the frequency of matching the indicated motifs among all sites detected in the indicated samples. Data information: Statistics: Fisher's exact test, *$P < 0.05$. (**B**) Analysing only phospho-sites detected in both *cdc28-as* and wild-type, pie charts of the proportion of no change, increased or decreased sites when comparing *cdc28-as* and wild type in either the meiosis I samples (left) or meiosis II samples (right). (**C**) Icelogo comparing phospho-sites decreased in *cdc28-as* vs wild type in meiosis I and sites that are not significantly changed. (**D**) Fisher tests comparing the enrichment of motifs in the indicated groups of phospho-sites (same groups as in pie chart in (**B**, left)). Data information: Statistics: Fisher's exact test, **$P < 0.01$. (**E**) Icelogo comparing phospho-sites decreased in *cdc28-as* vs wild type in meiosis II and sites that are not significantly changed. (**F**) Fisher tests comparing the enrichment of motifs in the indicated groups of phospho-sites (same groups as in pie chart in (**B**, right)). Data information: Statistics: Fisher's exact test, **$P < 0.01$, ***$P < 0.001$. Source data are available online for this figure.

© The Author(s)

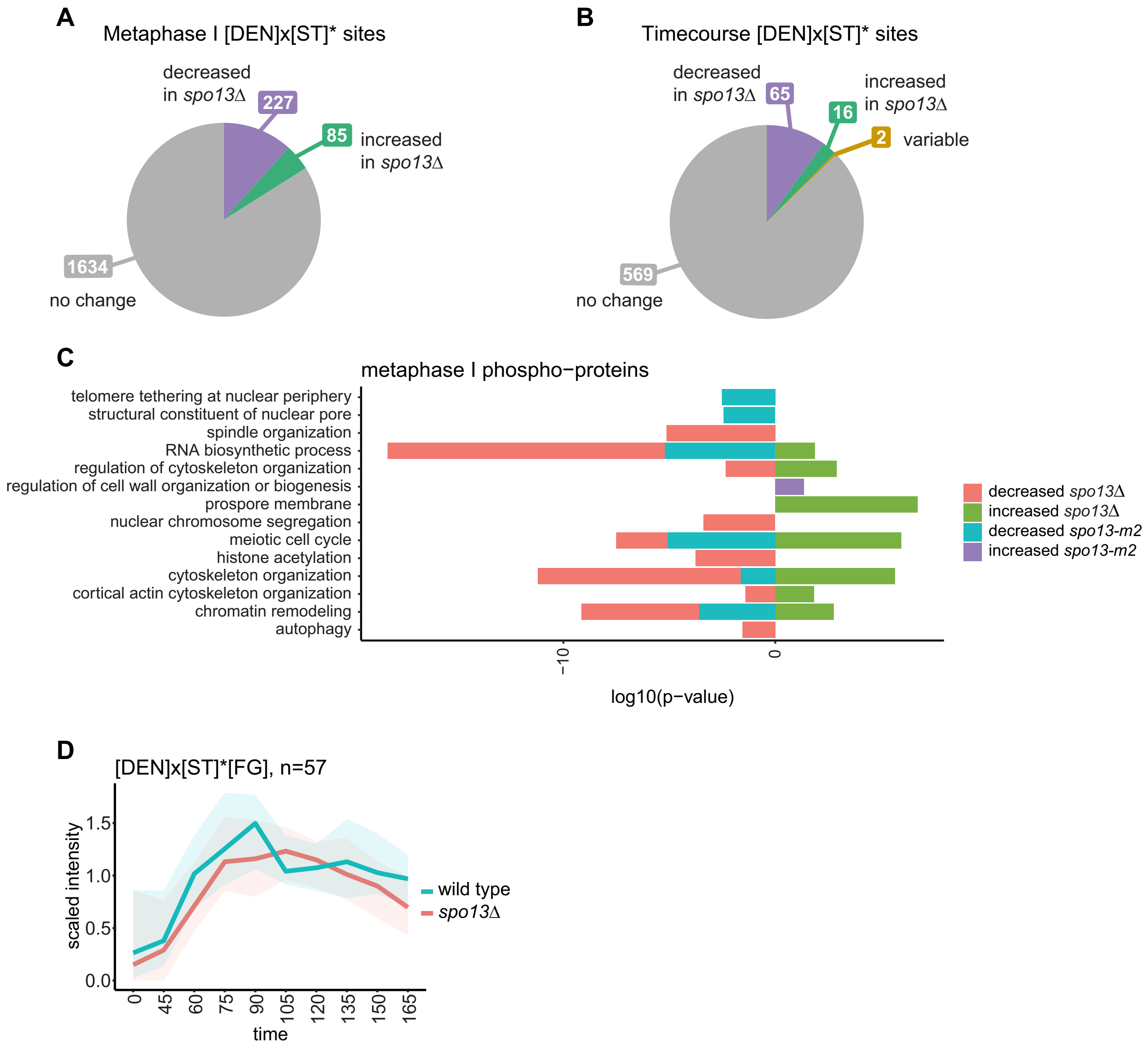

Figure EV5. Comparison of Cdc5[Polo] kinase motif phosphorylation between wild type and *spo13*Δ.

(**A**) Proportion of [DEN]x[ST]* motif-matching phospho-sites with significantly different abundance in *spo13*Δ versus wild type in metaphase I-arrested cells. (**B**) Proportion of [DEN]x[ST]* motif-matching phospho-sites with significantly different abundance in *spo13*Δ versus wild type in the meiotic timecourse experiments. (**C**) GO terms enriched among proteins with significantly different phosphorylation in *spo13*Δ or *spo13-m2* versus wild type. Data information: Statistics: Cumulative hypergeometric test followed by correction for multiple testing (gprofiler2 R package gost function default settings). (**D**) Abundance of phospho-sites matching the [DEN]x[ST]*[FG] motif among sites detected in both replicates of wild type and *spo13*Δ across the timecourse. Source data are available online for this figure.

 © The Author(s)

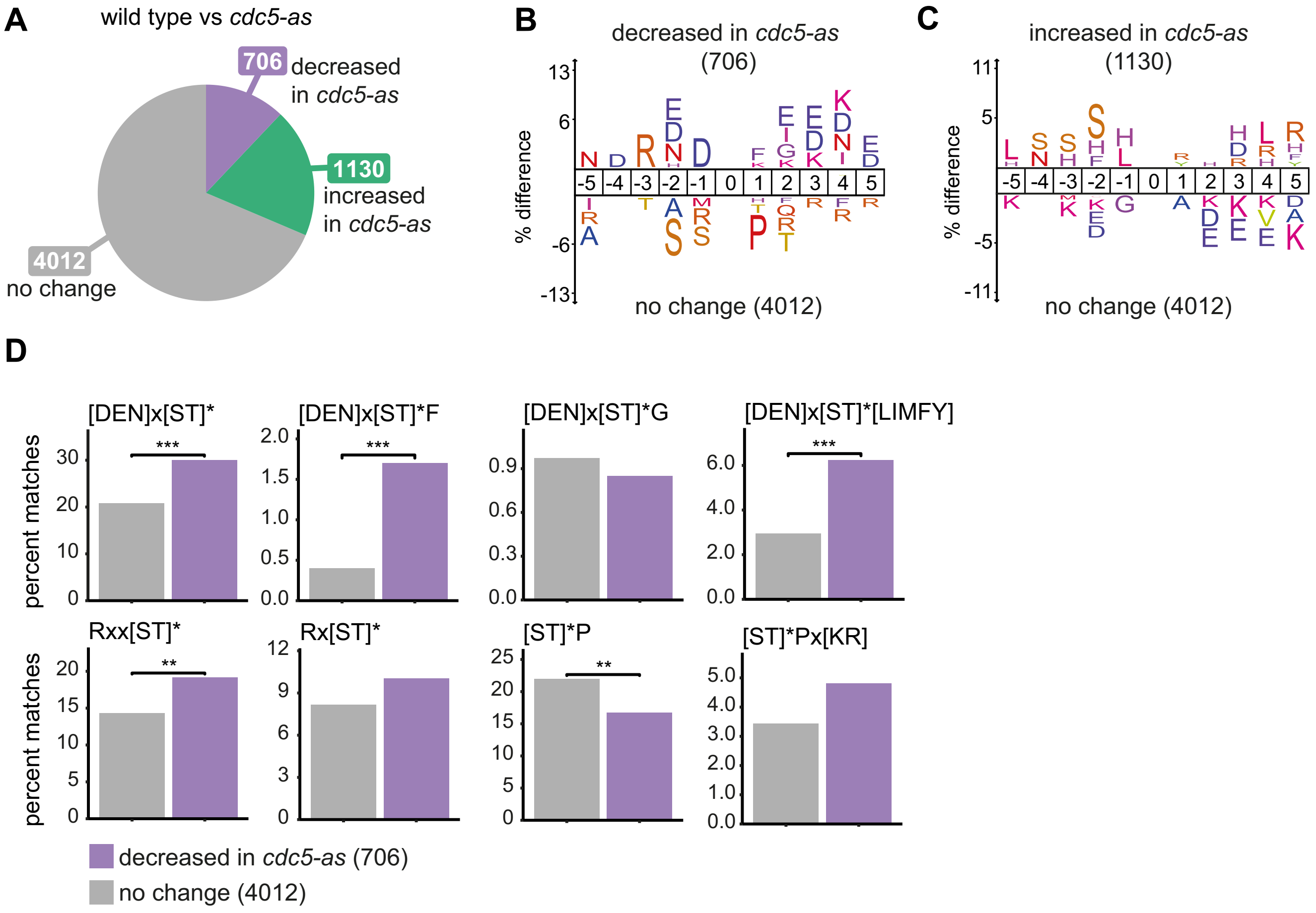

Figure EV6. [DEN]x[ST]* and [DEN]x[ST]*F motif phosphorylation depends on Cdc5^{Polo}.

(**A**) Pie chart showing the proportion of increased, decreased and no change sites between *cdc5-as* and wild type in prometaphase. (**B**) IceLogo comparing amino acid frequency between sites that were decreased in *cdc5-as* compared to no change sites. (**C**) IceLogo comparing amino acid frequency between sites that were increased in *cdc5-as* compared to no change sites. (**D**) Fisher tests comparing the number of the indicated motif-matching sites between the groups of sites that were decreased in *cdc5-as* (purple) or no change (grey). Data information: Statistics: Fisher's exact test, $^{**}P < 0.01$, $^{***}P < 0.001$. Source data are available online for this figure.
